# Transmission rates of the bacterial endosymbiont, *Neorickettsia risticii*, during the asexual reproduction phase of its digenean host, *Plagiorchis elegans*, within naturally infected lymnaeid snails

**DOI:** 10.1186/1756-3305-6-303

**Published:** 2013-10-22

**Authors:** Stephen E Greiman, Vasyl V Tkach, Jefferson A Vaughan

**Affiliations:** 1 Department of Biology, University of North Dakota, 10 Cornell St., Grand Forks, North Dakota 58202, USA

**Keywords:** *Neorickettsia*, Digenean, Transmission, Cercariae, Sporocysts

## Abstract

**Background:**

*Neorickettsia* are obligate intracellular bacterial endosymbionts of digenean parasites present in all lifestages of digeneans. Quantitative information on the transmission of neorickettsial endosymbionts throughout the complex life cycles of digeneans is lacking. This study quantified the transmission of *Neorickettsia* during the asexual reproductive phase of a digenean parasite, *Plagiorchis elegans*, developing within naturally parasitized lymnaeid pond snails.

**Methods:**

*Lymnaea stagnalis* snails were collected from 3 ponds in Nelson County, North Dakota and screened for the presence of digenean cercariae. Cercariae were identified to species by PCR and sequencing of the 28S rRNA gene. *Neorickettsia* infections were initially detected using nested PCR and sequencing of a partial 16S rRNA gene of pooled cercariae shed from each parasitized snail. Fifty to 100 single cercariae or sporocysts were isolated from each of six parasitized snails and tested for the presence of *Neorickettsia* using nested PCR to estimate the efficiency at which *Neorickettsia* were transmitted to cercariae during asexual development of the digenean.

**Results:**

A total of 616 *L. stagnalis* were collected and 240 (39%) shed digenean cercariae. Of these, 18 (8%) were *Neorickettsia*-positive. Six *Neorickettsia* infections were selected to determine the transmission efficiency of *Neorickettsia* from mother to daughter sporocyst and from daughter sporocyst to cercaria. The prevalence of neorickettsiae in cercariae varied from 11 to 91%. The prevalence of neorickettsiae in sporocysts from one snail was 100%.

**Conclusion:**

Prevalence of *Neorickettsia* infection in cercariae of *Plagiorchis elegans* was variable and never reached 100%. Reasons for this are speculative, however, the low prevalence of *Neorickettsia* observed in some of our samples (11 to 52%) differs from the high prevalence of other, related bacterial endosymbionts, e.g. *Wolbachia* in *Wolbachia*-dependent filariid nematodes, where the prevalence among progeny is universally 100%. This suggests that, unlike the *Wolbachia*-filaria relationship, the *Neorickettsia*-digenean relationship is not obligatory mutualism. Our study represents the first quantitative estimate of the *Neorickettsia* transmission through the asexual phase of the digenean life cycle.

## Background

**Figure 1:**
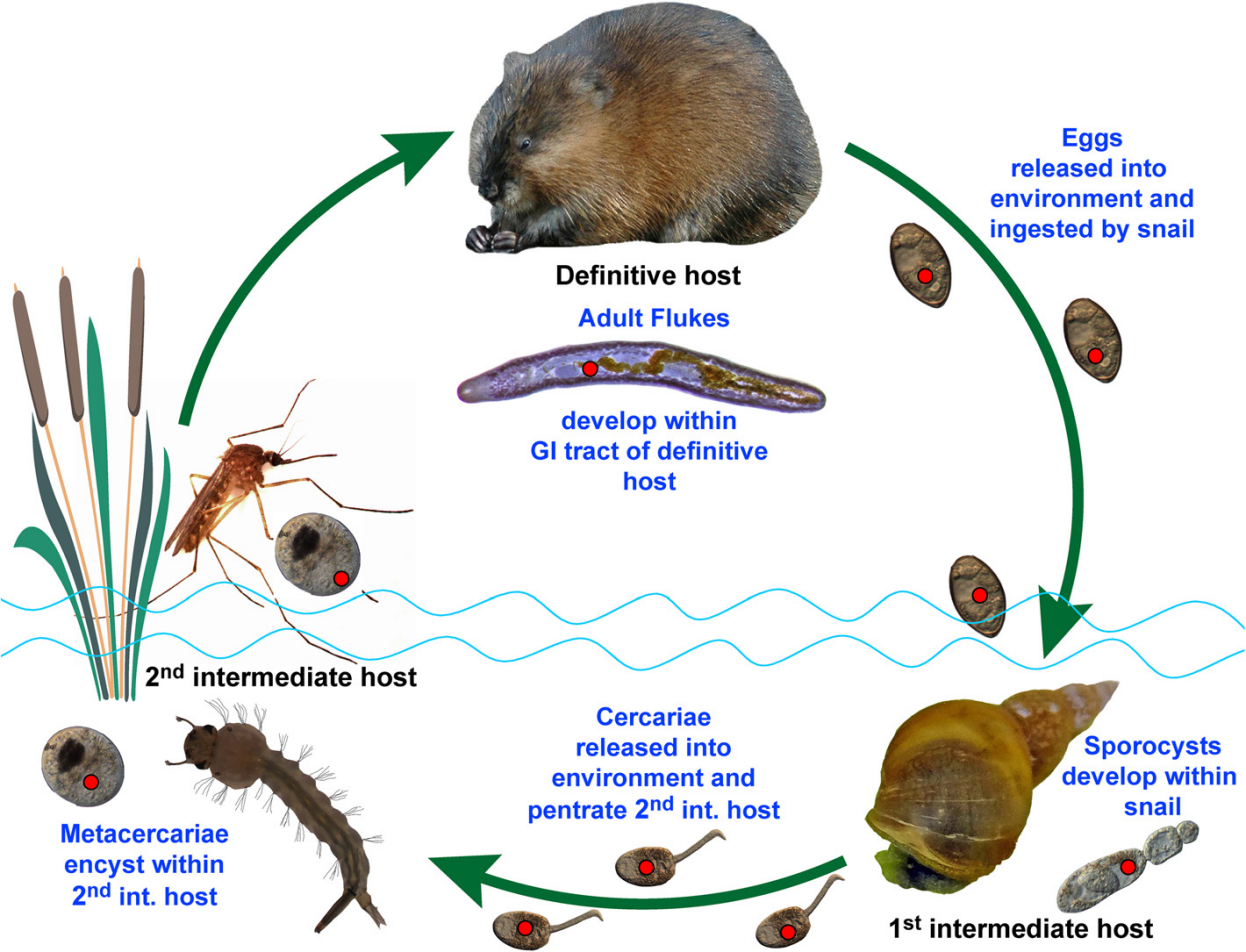
**Natural life cycle of the digenean *Plagiorchis elegans*. ***Neorickettsia* infection is represented by a red dot.

Bacteria in the genus *Neorickettsia* (Order Rickettsiales, Family Anaplasmataceae) are intracellular endosymbionts of digeneans. Neorickettsiae are presumably maintained throughout the digenean life cycle by vertical transmission during the sexual and asexual reproductive phases of the parasite. Digeneans are endoparasitic flatworms with complex life cycles involving asexual reproduction in mollusks (=first intermediate host) and sexual reproduction in vertebrates (=definitive host) (Figure 1). In some cases neorickettsiae are transmitted horizontally from digeneans to their vertebrate definitive hosts, where the bacteria can infect white blood cells and cause debilitating disease in horses, dogs, and humans [1-6]. Currently, *Neorickettsia* comprises three named species and several unnamed species level lineages [6,7].

*Neorickettsia risticii* is the most widespread member of the genus in the United States and worldwide [1,7-11]; for detailed review see [6]. It is known to cause a horse illness called equine monocytic ehrlichiosis or Potomac horse fever referring to the region where it was first discovered. Its clinical symptoms vary and usually include fever, depression, anorexia and colitis, accompanied by acute diarrhea [1,10]. If untreated the disease causes abortions in pregnant mares and relatively high mortality approaching 30% [12,13]. Due to the fact that *N. risticii* was initially placed in the genus *Ehrlichia*, the disease was first thought to be transmitted by ticks. However, as a result of combined molecular and experimental approaches it was demonstrated to be associated with digeneans [14-18]. Since then, *N. risticii* was detected in representatives of several digenean families, e. g., Lecithodendriidae, Plagiorchiidae, Microphallidae, Macroderoididae, and Echinostomatidae [7,17-25]. Although the digenean hosts of *N. risticii* have diverse life cycles that utilize various groups of invertebrates and even vertebrate intermediate hosts, it has been shown that amphibiotic insects such as mayflies and caddisflies play a particularly important role in the epizootology of Potomac horse fever [20,22,26]. When horses swallow insects infected by metacercariae harboring *Neorickettsia*, they may develop Potomac horse fever even though the digeneans cannot develop to adult stages in horses.

Despite their role as pathogens of humans, domestic animals and wildlife, practically no quantitative information exists concerning the transmission of *Neorickettsia* through the digenean life cycle. Gibson *et al.*[23] used immunofluorescence and PCR to demonstrate vertical transmission of *N. risticii* from the adult digenean *Acanthatrium oregonense* to its eggs and horizontal transmission of bacteria from the digeneans to their bat host. These authors randomly removed 5 individual eggs from each of two bats and screened them for *N. risticii* by PCR. Seven out of 10 eggs were positive for *Neorickettsia*, however, it was unclear whether each set of eggs originated from a single worm.

Ability to pass and reproduce through both sexual and asexual phases of the digenean life cycle is essential to the perpetuation of *Neorickettsia* in nature. This study reports the prevalence of *Neorickettsia* endosymbionts in cercariae (i.e., progeny of digenean asexual stages) of the digenean *Plagiorchis elegans* (family Plagiorchiidae) shed by naturally-parasitized pond snails *Lymnea stagnalis* collected in eastern North Dakota. *P. elegans* has a life cycle typical for the Plagiorchiidae that involves an aquatic snail (in this case *Lymnaea stagnalis*) as the first intermediate host, an arthropod as the second intermediate host and a vertebrate as the definitive host (Figure 1). After eggs are laid by an adult worm they are shed with host feces to the external environment. In water, eggs undergo embryonation and need to be ingested by a snail. The next stage, miracidium, hatches in the intestine of the snail, penetrates through the intestinal wall, reaches the hepatopancreas of the mollusk and develops into a mother sporocyst. The mother sporocyst goes through multiple rounds of asexual reproduction, producing a large number of daughter sporocysts. The daughter sporocysts produce a large number of the next stage, cercariae, through asexual reproduction. Cercariae are shed by the mollusk into the aquatic environment where they may penetrate an aquatic arthropod and encyst to form metacercaria. After some development, the metacercaria needs to be swallowed by an appropriate vertebrate host to mature into adult stage (Figure 1).

To the best of our knowledge, these data represent the first quantitative estimates of infection rates for *Neorickettsia* endosymbionts during the asexual reproductive phase of digenean parasites.

## Methods

*Lymnaea stagnalis* snails were collected during the summer months of 2011–2012 from 3 ponds in Nelson County, North Dakota; Pond 1 (Lat: 48°0′24.18″N and Long: 97°56′37.32″W), Pond 2 (48°1′43.80″N, 97°59′24.88″W), Pond 3 (48°10′48.60″N, 98°7′51.29″W). Snails were rinsed with water and placed individually into glass jars filled with aged tap water treated with commercial aquarium conditioner to remove chlorine/chloramines. Snails were kept for several hours under fluorescent lamps followed by several hours without light. Afterwards, the water in jars was examined for the presence of cercariae. Snails shedding cercariae were maintained individually in labeled containers and fed with lettuce.

Initial screening for *Neorickettsia* was carried out using pooled cercariae. Fifty cercariae from each shedding snail were pipetted into a 1.75 ml microcentrifuge tube and centrifuged at 13,000 rpm for five minutes. Supernatant was removed and 75 μl of ultrapure water added. Cercariae were disrupted by direct sonication using a UP100H compact ultrasonic processor (Hielscher USA, Inc., Ringwood, NJ) at an amplitude of 70% for 15 seconds, and immediately placed on ice to prevent DNA degradation due to enzymatic activity. Sonicates were used directly as a template for PCR amplification procedures. DNA was extracted from the remainder of each homogenate using the guanidine thiocyanate method [27] and used for the digenean taxon identification.

If *Neorickettsia* was detected in pooled cercariae from an individual snail, 50 or 100 individual cercariae or sporocysts from that snail were tested for *Neorickettsia*. One of the snails with *Neorickettsia*-positive digenean infection was dissected for individual screening of both sporocysts and cercariae. Individual sporocysts were carefully separated using very fine needles under high power of a stereo microscope. A single cercaria or sporocyst was placed in 50 μl of ultrapure water and disrupted by direct sonication as described above. We did not detect any PCR inhibition with either pooled cercarial or single cercarial/sporocyst sonicates.

**Figure 2:**
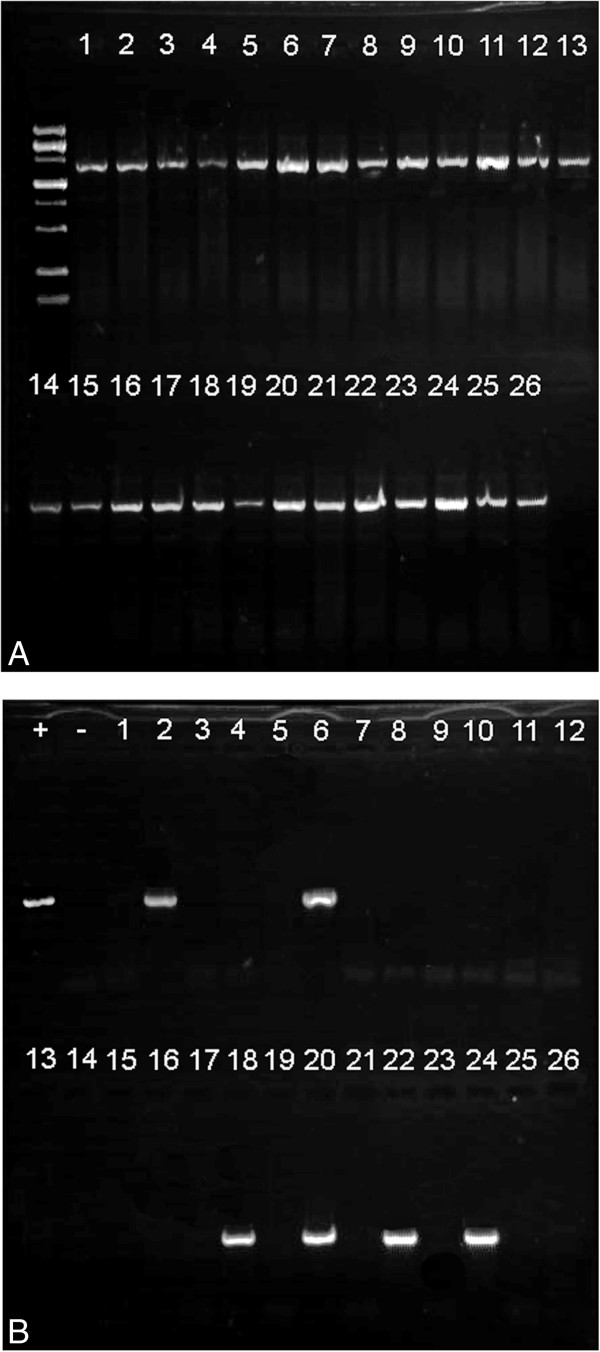
**Electrophoregrams showing results of PCR of bacterial and digenean DNA from the same extracts obtained from single cercariae. (A)** Results of amplification of digenean partial 28S gene from 26 single cercarial homogenates. **(B)** Results of screening the same 26 single cercarial homogenates for *Neorickettsia* DNA by nested PCR of partial 16S gene. Lanes with positive and negative controls are labeled.

To validate the use of sonication as a method of obtaining DNA from single cercariae, 26 single cercariae were sonicated, screened for neorickettsial DNA using a nested PCR protocol as well as for digenean DNA using digenean-specific primers with a standard PCR protocol. All sonicates produced good amplicons of digenean DNA indicating that sonication of single cercariae yielded sufficient quantity and quality of DNA for use in PCR (Figure 2).

Sonicates were assayed for *N. risticii* using a substantially modified nested PCR protocol initially described by Barlough *et al*. [28]. Five microliters of each sonicate were used for the first PCR reaction and 1 μl (pooled cercariae) or 3 μl (single cercariae/sporocysts) of the first PCR product were used for the nested PCR. The nested PCR amplified a 527-bp portion of the 5″ end of the 16S rRNA gene. The primer pairs (designed by SEG) used in the first round were n16S-25 F (5′-TCAGAACGAACGCTAGCGGT-3′) and n16S-610R (5′-GACGTTCCTCTTGATATCTACG-3′). Primers used in the nested PCR round were n16S-70 F (5′-GAATCAGGGCTGCTTGCA-3′; designed by SEG) and ER2-R (5′-GTTTTAAATGCAGTTCTTGG-3′ from Barlough *et al*. [28]).

The PCR reactions were run on an EP Gradient thermocycler (Eppendorf, Hauppauge, NY) using Quick load OneTaq mastermix (New England Biolabs, Ipswich, MA) according to the manufacturer’s instructions. Annealing temperature of 54°C and 40 cycles were used in both initial and nested PCRs. DNA extracts of *N. risticii* were used as positive controls (graciously provided by Dr. John Madigan, UC Davis). In negative controls, ultrapure water was used in place of sonicates.

Digenean cercariae were identified to species using partial sequences of the 28S rRNA gene. Digenean DNA was amplified by PCR using forward primer digl2 (5′-AAGCATATCACTAAGCGG-3′) and reverse primer 1500R (5′-GCTATCCTGAGGGAAACTTCG-3′) designed by VVT.

PCR amplicons of both *Neorickettsia* and digeneans were purified using either the DNA Clean & Concentrator™ -5 kit (Zymo Research, Irvine, CA) or ExoSap PCR clean-up enzymatic kit (Affimetrix, Santa Clara, CA), according to the manufacturers’ instructions. The PCR products were cycle-sequenced using ABI BigDye™ chemistry, alcohol precipitated and run on an ABI Prism 3100™ automated capillary sequencer. PCR products were sequenced in both directions. For sequencing of *Neorickettsia*, nested PCR primers were used. For sequencing of cercariae, the PCR primers as well as internal forward primers 300 F (5′-CAAGTACCGTGAGGGAAAGTTG-3′), 900 F (5′-CCGTCTTGAAACACGGACCAAG-3′) and internal reverse primers 300R (5′-CAACTTTCCCTCACGGTACTTG-3′) and ECD2 (5′-CTTGGTCCGTGTTTCAAGACGGG-3′) were used. Contiguous sequences of *Neorickettsia* and cercariae/sporocysts were assembled and edited using Sequencher™ ver. 4.2 (GeneCodes Corp., Ann Arbor, MI) and submitted to GenBank under accession numbers: *Plagiorchis elegans* [GenBank: KF556678] and *Neorickettsia risticii* [GenBank: KF556679].

## Results

Out of 616 *L. stagnalis* collected in all three ponds, 240 shed digenean cercariae (Table 1). Eighteen (8%) of the 240 cercarial infections were *Neorickettsia*-positive. Prevalences of *Neorickettisia* in single cercariae and sporocysts were only studied from Pond 2. This pond had the highest prevalence of *Neorickettsia* (23%) in digeneans, although not the highest prevalence of digeneans in snails (Table 1). Using DNA sequencing, we were able to identify both the digenean and the neorickettsial endosymbiont. All *Neorickettsia*-positive infections represented a single digenean species, a plagiorchiid *Plagiorchis elegans*. Sequences of cercariae were identical to the sequence of adult *P. elegans* from the same area published by Tkach *et al.*[7].

**Table 1:** **Infection prevalences for digeneans in snails and *Neorickettsia *** **in digeneans in Nelson Co., North Dakota**

	**Prevalence of digeneans = % in snails (N)**	**Prevalence of *Neorickettsia*** **= % among snails infected with digeneans (N)**
Pond 1	9% (100)	11% (9)
Pond 2	25% (187)	23% (47)
Pond 3	56% (329)	3% (184)
Total	39% (616)	8% (240)

We used 5 *P. elegans* infections for analysis of the efficiency of *Neorickettsia* transmission through the asexual stage of the digenean life cycle. All 5 samples of *P*. *elegans* harbored *Neorickettsia risticii*, the agent of the Potomac horse fever. The prevalence of individually screened cercariae infected with *Neorickettsia* varied from 11% to 91% among the 5 digenean samples (Table 2). Fifty sporocysts and 50 cercariae were screened from one of the snails. In this case, all sporocysts assayed were positive for *N. risticii*, but only 90% of cercariae from the same snail had neorickettsial infection (Table 2; χ^2^ with Yates correction = 3.37, df = 1, p = 0.07).

**Table 2:** **Prevalence of *Neorickettsia *** **infection among individual cercariae and sporocysts parasitizing snails, Nelson Co., North Dakota**

**Digenean species**	**Life cycle stage**	**% positive (N)**
*Plagiorchis elegans*	Cercariae	11% (100)
*Plagiorchis elegans*	Cercariae	52% (100)
*Plagiorchis elegans*	Cercariae	70% (50)
*Plagiorchis elegans*	Cercariae	91% (100)
*Plagiorchis elegans*	Cercariae*	90% (50)
*Plagiorchis elegans*	Sporocysts*	100% (50)

*sporocysts and cercariae obtained from the same snail individual.

## Discussion

Transmission of *Neorickettsia* through the complex digenean life cycle does not fit easily into the classical categories of vertical transmission. Due to the presence of both sexual and asexual stages in digenean life cycles it is clear that the vertical transmission of *Neorickettsia* through the digenean life cycle is not wholly dependent on transovarian transmission as in the case of some other bacterial pathogens/symbionts of invertebrates.

Although all 3 ponds surveyed in the study area produced some snails harboring *Neorickettsia*-infected digeneans (Table 1), the prevalence of *Neorickettsia* infection between ponds differed dramatically, from only 3.3% in Pond 3 to 23.4% in Pond 2. At the same time, the highest prevalence of digenean infections in snails was in Pond 3, reaching 56% vs 25% in Pond 2 (Table 1). This illustrates the highly heterogenous nature of *N. risticii* distribution within a landscape and has certain implications for the epidemiology of Potomac horse fever caused by this bacterial species.

It is noteworthy that both low and high prevalences of neorickettsial infections of individual cercaria were found in the same parasite species (*P. elegans*), the same snail species (*L. stagnalis*), and the same body of water (Pond 2; Table 2). Thus, the observed differences in neorickettsial prevalence in single cercariae were not correlated with the digenean host, snail host, or locality. Overall, our data suggest that there is a lot of inherent variability in the efficiency of vertical transmission of *Neorickettsia* during asexual reproduction of digeneans. The reasons for this variability are currently unknown and may depend on a number of factors. Some of the possible explanations are provided below.

**Figure 3:**
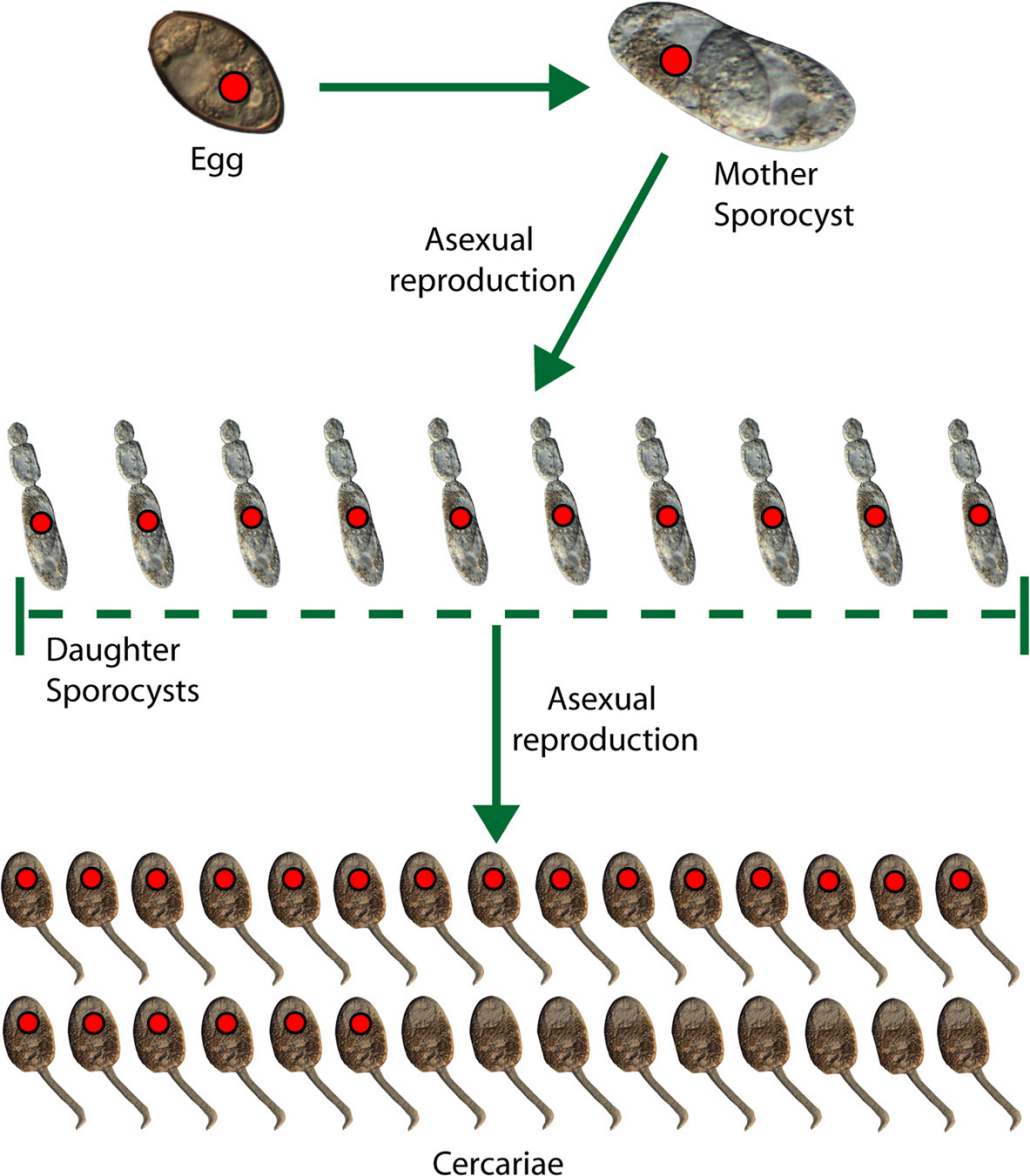
**Transmission efficiency of *Neorickettsia *through asexual stages of *P. elegans *life cycle.** Infection with *Neorickettsia* is represented by a red dot.

Our data on the prevalence of neorickettsial infection in sporocysts and their cercarial progeny from the same snail (Table 2) indicate that most, but not all, progeny produced by an infected sporocyst inherit the bacterial endosymbiont (Figure 3). Perhaps, the initial intensity of *Neorickettsia* infection in egg and/or sporocyst may determine the proportion of cercarial progeny inheriting neorickettsial endosymbionts. For example, a sporocyst infected with high bacterial load may transmit *Neorickettsia* to a larger proportion of its cercarial progeny than a sporocyst with fewer bacteriae. This “dosage effect” would be similar to what has been described in the transovarial infection of ticks with *Rickettsia rickettsii*. Female ticks with high intensity of infection transmitted rickettsiae to 100% of their progeny, while those with mild infections produced considerably lower percentages of infected eggs [29].

Alternatively, the rate of neorickettsial replication may be slower than the rate of development and replication of digenean asexual stages. If true, this asynchrony could result in a lower *Neorickettsia* prevalence in cercariae produced by snails that have only recently begun shedding compared to snails with older digenean infection.

Transmission in the digenean-*Neorickettsia* system is different from that of the other members of the family Anaplasmataceae symbiotic with invertebrates. The Anaplasmataceae includes 3 or 4 (depending on the systematic views of different authors) other genera associated with either ticks or other arthropods (*Ehrlichia*, *Anaplasma*, *Wolbachia*) or filarial nematodes (*Wolbachia*). In the case of *Ehrlichia* and *Anaplasma*, vertical transmission plays little role in their circulation [5,30]. In contrast, *Wolbachia* infects the ovaries and other tissues of many species of arthropods and filarial nematodes and is transmitted exclusively by vertical transmission [31-33]. Thus, *Neorickettsia* are unique among the members of the family because it can be transmitted both vertically and horizontally. In this respect, the patterns of transmission exhibited by *Neorickettsia* are more similar to those observed among pathogenic spotted fever group *Rickettsia* (e.g., *R. rickettsii*, family Rickettsiaceae, order Rickettsiales) [34].

Like *Neorickettsia*, *Wolbachia* is maintained through vertical transmission, however, there are key differences between the 2 genera of bacteria. *Wolbachia* and the filariae have a mutualistic relationship [35-37]. Ferri *et al.*[32] supposed that the bacteria may be essential to the biosynthesis of some molecules necessary for filarial host fertility and viability, such as heme, riboflavin or nucleotide synthesis. As a result, filarial species that host *Wolbachia*, are dependent on the presence of these endosymbionts. Landmann *et al*. [33] showed that filarial nematodes infected with *Wolbachia* pass it on to 100% of eggs. The exact nature of the interrelationships between *Neorickettsia* and their digenean hosts is not known. Currently, there is no evidence suggesting a mutualistic relationship between digeneans and *Neorickettsia*. In our study, the majority of *P. elegans* did not harbor *Neorickettsia*, which proves that these digeneans are not dependent on their neorickettsial endosymbionts. Presently available data are insufficient to classify the endosymbiotic relationships of *Neorickettsia* with digeneans as either parasitic or commensal.

## Conclusions

Our data demonstrate that the transmission efficiency of *Neorickettsia* through asexual stages of the *P. elegans* life cycle is lower than 100% (Figure 3). In cercariae from naturally infected snails, the prevalence of *Neorickettsia* ranged from 11 to 91%. Even in a case where 100% of screened daughter sporocysts harbored *Neorickettsia*, only 90% of the cercarial progeny were infected. These findings are in contrast with the situation in some other bacterial endosymbionts, such as *Wolbachia* in *Wolbachia*-dependent filariid nematodes, where the vertical transmission rates are 100%.

## Competing interests

The authors declare that they have no competing interests.

## Authors’ contributions

SEG participated in study design, collected snails, analyzed the data, carried out experiments, created figures, and drafted the manuscript. VVT participated in study design, collected snails, analyzed the data, and helped draft the manuscript. JAV participated in study design, analyzed the data, and helped draft the manuscript. All authors read and approved the final version of the manuscript.
